# Whole genome transcript profiling from fingerstick blood samples: a comparison and feasibility study

**DOI:** 10.1186/1471-2164-10-617

**Published:** 2009-12-17

**Authors:** Elizabeth H Robison, Tony S Mondala, Adam R Williams, Steven R Head, Daniel R Salomon, Sunil M Kurian

**Affiliations:** 1 DNA Array Core, The Scripps Research Institute, La Jolla, CA, 92037, USA; 2 Department of Molecular and Experimental Medicine, The Scripps Research Institute, La Jolla, CA, 92037, USA; 3 Transplant Genomics Collaborative Group, The Scripps Research Institute, La Jolla, CA, 92037, USA

## Abstract

**Background:**

Whole genome gene expression profiling has revolutionized research in the past decade especially with the advent of microarrays. Recently, there have been significant improvements in whole blood RNA isolation techniques which, through stabilization of RNA at the time of sample collection, avoid bias and artifacts introduced during sample handling. Despite these improvements, current human whole blood RNA stabilization/isolation kits are limited by the requirement of a venous blood sample of at least 2.5 mL. While fingerstick blood collection has been used for many different assays, there has yet to be a kit developed to isolate high quality RNA for use in gene expression studies from such small human samples. The clinical and field testing advantages of obtaining reliable and reproducible gene expression data from a fingerstick are many; it is less invasive, time saving, more mobile, and eliminates the need of a trained phlebotomist. Furthermore, this method could also be employed in small animal studies, i.e. mice, where larger sample collections often require sacrificing the animal. In this study, we offer a rapid and simple method to extract sufficient amounts of high quality total RNA from approximately 70 μl of whole blood collected via a fingerstick using a modified protocol of the commercially available Qiagen PAXgene RNA Blood Kit.

**Results:**

From two sets of fingerstick collections, about 70 uL whole blood collected via finger lancet and capillary tube, we recovered an average of 252.6 ng total RNA with an average RIN of 9.3. The post-amplification yields for 50 ng of total RNA averaged at 7.0 ug cDNA. The cDNA hybridized to Affymetrix HG-U133 Plus 2.0 GeneChips had an average % Present call of 52.5%. Both fingerstick collections were highly correlated with r^2 ^values ranging from 0.94 to 0.97. Similarly both fingerstick collections were highly correlated to the venous collection with r^2 ^values ranging from 0.88 to 0.96 for fingerstick collection 1 and 0.94 to 0.96 for fingerstick collection 2.

**Conclusions:**

Our comparisons of RNA quality and gene expression data of the fingerstick method with traditionally processed sample workflows demonstrate excellent RNA quality from the capillary collection as well as very high correlations of gene expression data.

## Background

Whole genome gene expression profiling has revolutionized research in the past decade especially with the advent of DNA microarrays [1-3]. This versatile technique has facilitated the parallel interrogation of thousands of RNA transcripts, simultaneously from a variety of tissues. Keeping up with the pace of development of DNA microarrays are several methods of RNA isolation from tissues and cells, the critical first step in the generation of data. More recently, to avoid bias as well as manipulation of blood cells during their processing, there are now a few commercial kits available for whole blood RNA isolation and purification such as the PAXgene system (Qiagen, Valencia, CA) and the Tempus Blood RNA collection and Isolation system (Applied Biosystems, Foster City, CA). These methods require at least 2.5 mL blood via venipuncture that is collected into a tube containing proprietary RNA stabilizing reagents that simultaneously lyses the whole blood as well as stabilizes the RNA at the time of collection thereby immobilizing and preventing further changes to the RNA transcriptome. This method has been imperative for the generation of reliable gene expression data from whole blood where the RNA is highly susceptible to changes and degradation during subsequent manipulation of the blood cells creating significant artifacts of sample collection and processing, especially when drawn in clinical situations [4]

However, one limitation of such RNA stabilization systems in humans is the requirement for the collection of a venous blood sample, which involves a venipuncture. Fingerstick capillary blood collection has been widely used and has been shown to be very reliable. A classic example of this methodology is blood glucose monitoring. Fingerstick blood has also been used to test for *Helicobacter pylori *infection [5,6], cholesterol [7,8], glycosylated hemoglobin (A1c) levels [9] and syphilis [9]. However, a review of the current literature reveals that there are no protocols available for extracting sufficient amounts of good quality total RNA from much smaller amounts of starting material for use in DNA microarray studies, specifically a droplet of blood from a finger stick (50-100 ul). A recent study used small volumes of blood (0.3mL was the smallest sample) along with a modified PAXgene protocol to obtain high quality RNA from paediatric samples, but they still obtained their samples via venipuncture [10]. Another recent study used small volumes (50 - 500μL) of mouse or rat blood, also with a modified PAXgene protocol, to obtain high quality RNA in sufficient amounts for microarray analysis [11]. In addition, there is a new Qiagen RNeasy Protect Animal Blood System protocol which promises to deliver high quality total RNA from 100 or 500 μL aliquots of rat or mouse blood. However, neither of these protocols were tested for use with human blood. The currently available Microtainer tubes from BD Biosciences are used for larger volumes (250-500 μL) and have no RNA stabilizing reagents making them unsuitable for DNA microarray studies, particularly for peripheral blood samples.

Obtaining reliable and reproducible gene expression data from a fingerstick has obvious advantages in clinical as well as field testing applications. A fingerstick is arguably a less invasive, less time consuming and a more mobile method of blood sample collection, eliminating the need of a trained phlebotomist. The utility of such a method is obvious in studies designed to collect blood samples from physically active subjects (i.e. soldiers and athletes) or for field studies in remote and under-developed areas. Fingerstick blood collection would also be of immense value in several types of subjects where it is commonly difficult to collect venous blood via venipuncture: infants and young children, intravenous drug addicts, and very obese individuals. The value of fingerstick capillary collection as opposed to venipuncture can also be appreciated in study designs where there is a need for serial sample collections or for pharmacokinetic studies that involve gene expression assays. This method could also be employed in small animal studies or nonhuman primates. Therefore, a fingerstick method of blood collection will broaden the current range of genomic profiling possible. We also believe that it will be an integral part of diagnosis and serial monitoring of disease states in the future where one can envision a hand-held device that could monitor expression levels of validated panels of genomic biomarkers, much like the glucose monitoring systems of today.

In the present study, we offer a rapid and simple method to extract sufficient amounts of high quality total RNA from approximately 70 μl of whole blood collected via fingerstick using a modified protocol of the commercially available Qiagen PAXgene RNA Blood Kit. RNA amplification, labeling, and fragmentation were performed using the Nugen Ovation kits (Nugen, San Carlos, CA). This approach hybridizes biotinylated cDNA onto the microarray and has been shown to perform with superior sensitivity especially with smaller amounts of input RNA [12-14]. Our comparisons of RNA quality and gene expression data with traditionally processed sample workflows demonstrate excellent RNA quality from the capillary collection as well as very high correlations of gene expression data in comparisons of venous and capillary blood collections.

## Results

In order to test the success and reproducibility of the fingerstick method of RNA isolation we took two fingerstick capillary samples from 5 donors on two separate days (four samples total from each donor, 20 total samples). From each collection time point we chose one purified total RNA sample from each donor and went forward with the RNA amplification, fragmentation and hybridization protocols. A venous sample was also collected in parallel from each of the donors by a trained phlebotomist and processed according to standard protocols to represent the established method http://www.scripps.edu/researchservices/dna_array[15]. All samples were assayed on Affymetrix HGU133 2.0 GeneChips using the Nugen standard array protocol for cDNA hybridization (Nugen FL-Ovation™ cDNA Biotin Module V2 - now called the Encore™ Biotin Module - user guide, http://www.nugeninc.com/tasks/sites/nugen/assets/File/user_guides/userguide_encore_biotin.pdf[16].

### Sample Collection, RNA Isolation, and Purification - Fingerstick Method

Approximately 70 μL of blood collected via fingerstick was combined with 200 μL PAXgene RNA stabilizing reagent (a PAXgene Reagent:blood ratio of 2.86) and left to incubate at room temperature for at least two hours as per manufacturer suggestions (PAXgene Blood RNA Kit handbook, http://www1.qiagen.com/Products/RnaStabilizationPurification/DSP/PaxGeneBloodRnaKitIVD.aspx#Tabs=t2[17].

We followed the PAXgene Blood RNA kit (product# 762164) protocol for RNA isolation and purification, with the exception of one modification. After the first spin, we washed the pellet with 1 mL RNase free water instead of 4 mL due to its small volume. We initially tested a "scaled down" version of the entire PAXgene protocol, but, through further testing, we found that using the standard volumes of buffers and washes had no effect on the yields and were easier to employ (data not shown). Furthermore, we also found the DNase step in the protocol was crucial for the yield and fidelity of the amplified cDNA. Without the DNase step, contaminating DNA was subsequently amplified causing the GAPDH and Actin ratios used as quality control metrics on the GeneChips to be abnormally high (data not shown).

### RNA Yield, Purity and Integrity - Fingerstick Method

From 20 samples of 70 μL fingerstick blood, the average total RNA yields ranged from 138 to 430 ng (average of 255.7 ± 72.6 ng) which was well above the maximum of 50 ng required for the Nugen Ovation RNA Amplification System v2 (Nugen, San Carlos, CA). While we did experience higher than normal OD_260/280 _ratios, an average of 3.9 ± 1.35, and lower OD_260/230 _ratios, an average of 0.09 ± .07, we found that this did not affect downstream applications and was probably caused by the high concentration of salts in the elution buffer relative to the low concentration of RNA http://www.flychip.org.uk/protocols/gene_expression/rna_qc.php[18]. To assess the quality of the RNA, the samples were then run on the Agilent 2100 BioAnalyzer using an RNA PicoChip. Due to sample anomalies identified by the software, two of the 20 samples were unable to be assigned a RNA Integrity Number (RIN); however, the traces looked normal and this appeared to have no affect on downstream applications for these two samples. The RIN numbers of the RNA from the remaining 18 samples were between 8.9 and 9.9 (average of 9.3 ± 0.25), which indicates RNA of high quality and integrity [19].

### RNA Yield, Purity and Integrity - Venipuncture Method

The total RNA from the 5 normal venipuncture PAXgene blood collection tubes was extracted and purified according to the PAXgene Blood RNA kit (product# 762164) protocol. From 2.5 mL of blood the yield ranged from 4.1 to 7.9 μg of total RNA (average 5.96 ± 1.5 μg) with an average OD_260/280_ratio of 2.0 ± 0.03 and an average OD_260/230 _ratio of 1.0 ± 0.45. To assess the quality and integrity of the RNA, the samples were then run on the Agilent 2100 BioAnalyzer using an RNA NanoChip. The average RIN of the 5 total RNA samples obtained by venipuncture was 9.2 ± 0.09.

### RNA Amplification, Labeling, and Fragmentation

**Figure 1:**
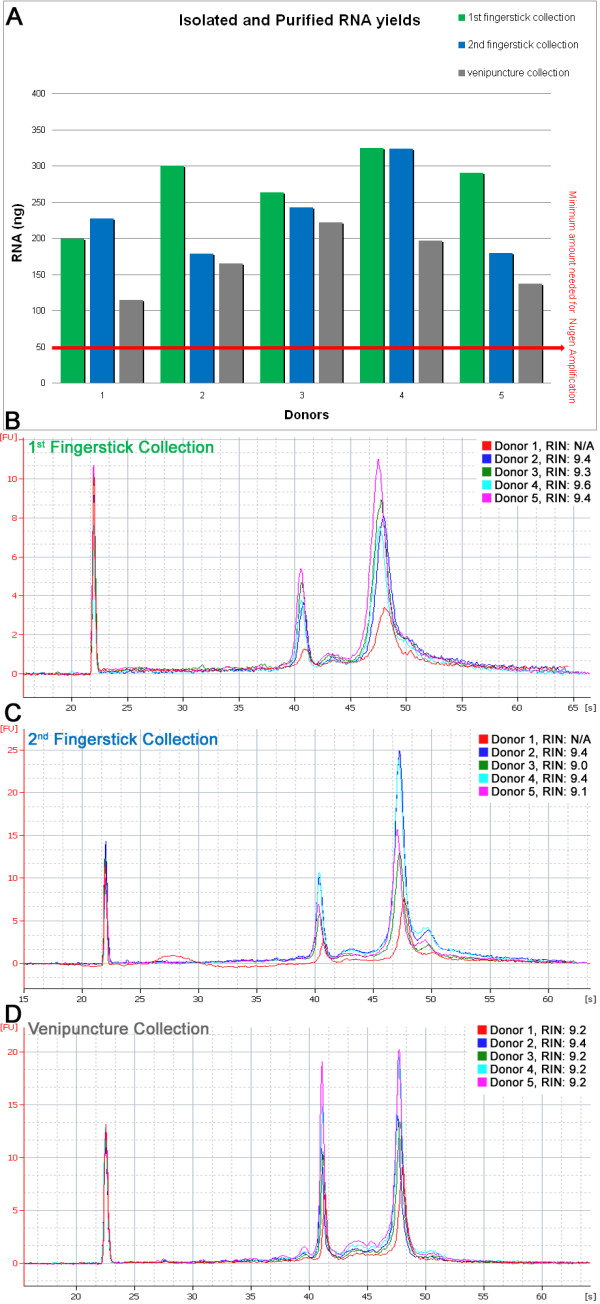
**Fingerstick and venipuncture RNA yields and Agilent 2100 bioanalyzer traces including RNA integrity numbers (RINs)**. **(A) **Fingerstick starting material: 70 uL, venipuncture starting material: 2.5 mL, yields normalized to 70 uL. **(B, C, D) **Agilent 2100 Bioanalyzer total RNA traces using the PicoChip **(B & C) **and the NanoChip **(D)**.

**Figure 2:**
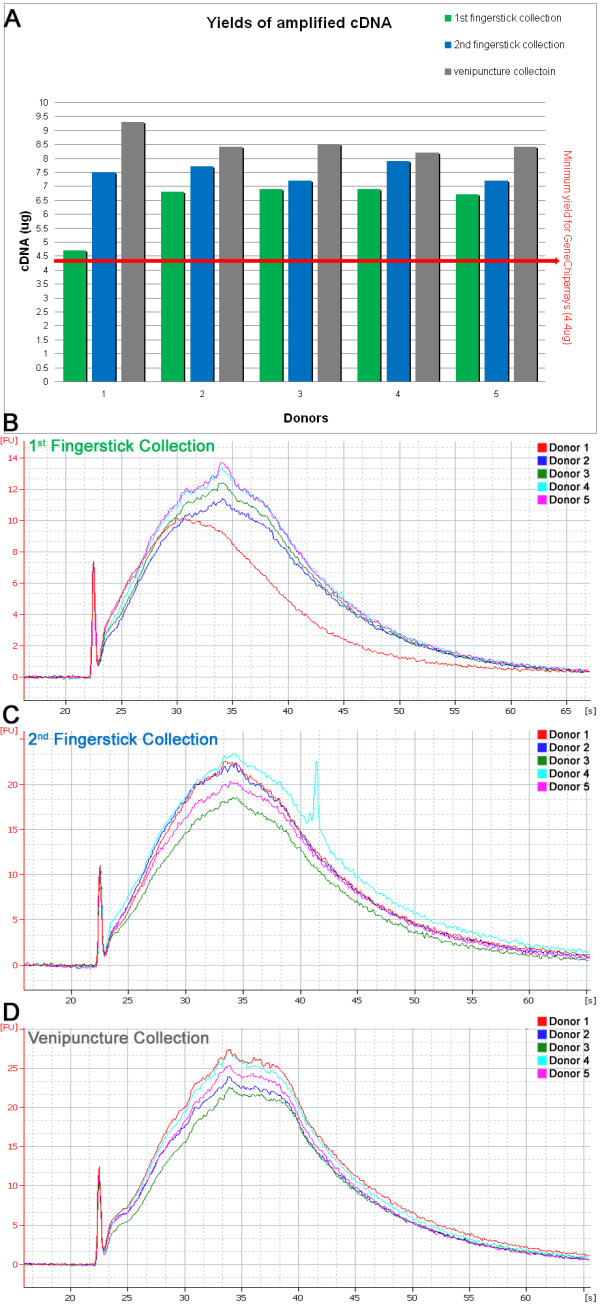
**Fingerstick and venipuncture cDNA yields and Agilent 2100 Bioanalyzer traces**. The samples included in this figure refer to the same samples as Figure 1. **(A) **Yields of cDNA post Nugen Ovation amplification of 50 ng total RNA. Yields of cDNA from both fingerstick and venipuncture samples were always above the 4.4 ug cut-off needed to continue with hybridization to GeneChip **(B, C, D) **Agilent 2100 Bioanalyzer cDNA traces using the NanoChip.

50 ng total RNA from each donor was taken from all three sample sets (fingerstick 1, fingerstick 2, and venipuncture) and subsequently amplified using the Nugen Ovation^® ^RNA Amplification System V2 (Cat.# 3100) and Ovation^® ^WB Reagent (Cat.# 1300). The total RNA yields and Agilent traces for these 15 samples are shown in Figure 1. After amplification, the samples were purified according to the Nugen user guide instructions using the Qiagen QIAquick^® ^PCR Purification Kit (Qiagen, Cat. #28104). As shown in Figure 2, the two batches of fingerstick samples (10 total) had cDNA yields ranging from 4.7 to 7.9 μg (average of 7.0 ± 0.88 μg), all satisfying the minimum requirement of 4.4 μg cDNA needed for Affymetrix chipping. The average OD_260/280_ratio of the 10 samples was 1.9 ± 0.01.

For the venipuncture samples (5 total), the cDNA yields ranged from 8.2 to 9.3 μg (average of 8.6 ± 0.43 μg) (Figure 2) with an average OD_260/280 _ratio of 1.9 ± 0.01. Before fragmenting and labeling the cDNA with the Nugen FL-Ovation™ cDNA Biotin Module V2 (Cat.# 4200), the BioAnalyzer was used again to determine quality of the amplified whole cDNA (Figure 2) and the results showed that all three sample sets had a consistent profile and size distribution across the RNA input range, indicating cDNA of good quality for array hybridization [Nugen Ovation RNA Amplification System v2 Technical Report #1, http://www.nugeninc.com/tasks/sites/nugen/assets/File/technical_documents/techdoc_ov_ampv2_rep_01.pdf[20].

### cDNA Hybridization and GeneChip Processing

4.4 μg of the labeled cDNA was then hybridized to Affymetrix GeneChip^® ^Human Genome U133 Plus 2.0 Arrays using the Nugen standard array protocol for cDNA hybridization (Nugen user guide). Following hybridization, the chip was then washed, stained and scanned according to standard Affymetrix protocol http://www.affymetrix.com/support/downloads/manuals/expression_analysis_technical_manual.pdf[21].

### GeneChip Data Quality Control

The Affymetrix quality metrics for the fingerstick samples are given in Table 1 and all the Venipuncture samples in Table 2. Both the fingerstick and the venous samples had similar average background (30.8 vs. 29.5) and scale factor (2.7 vs. 2.8). The average % present calls were slightly higher for the venipuncture samples (56% vs. 52.5%). The fingerstick samples had higher 3'/5' GAPDH and β-Actin ratios on average.

**Table 1:** Affymetrix quality metrics for all fingerstick samples.

Donor	Collection	Scale Factor	Background	% Present	GAPDH 3'/5'	B-actin 3'/5'
**1**	Fingerstick 1	4.51	31.1	44.7	32.2	158.6

**1**	fingerstick 2	2.76	29.9	52.4	7.7	29.4

**2**	fingerstick 1	2.58	31.9	52.8	6.6	13.3

**2**	fingerstick 2	2.25	32.1	54.2	6.2	19.7

**3**	fingerstick 1	2.70	30.8	52.7	4.3	6.6

**3**	fingerstick 2	2.23	30.2	54.3	5.8	15.0

**4**	fingerstick 1	2.77	31.2	53.1	5.3	8.8

**4**	fingerstick 2	2.13	30.2	53.8	6.3	16.8

**5**	fingerstick 1	2.82	29.7	53.3	4.9	6.9

**5**	fingerstick 2	2.25	30.9	53.5	7.5	19.1

**Average**		**2.7**	**30.8**	**52.5**	**8.7**	**29.4**

**SD**		**0.7**	**0.8**	**2.8**	**8.3**	**45.9**

**Table 2:** Affymetrix quality metrics for all venipuncture samples.

Donor	Collection	Scale Factor	Background	% Present	GAPDH 3'/5'	B-actin 3'/5'
**1**	venipuncture	3.10	30.3	56.9	2.9	1.9

**2**	venipuncture	2.82	29.4	56.4	3.0	1.7

**3**	venipuncture	3.15	29.0	56.2	2.3	1.7

**4**	venipuncture	2.52	30.1	56.6	3.0	1.8

**5**	venipuncture	2.63	28.4	54.1	2.8	1.7

**Average**		2.8	29.5	56.0	2.8	1.8

**SD**		0.3	0.8	1.1	0.3	0.1

### Correlation Coefficients for Fingerstick and Venipuncture Samples

We calculated correlation coefficients for each donor comparing fingerstick collection 1 vs. fingerstick collection 2 and also each fingerstick collection vs. the venipuncture collection (Table 3). Both fingerstick collections were highly correlated with r^2 ^values ranging from 0.94 to 0.97. Similarly both fingerstick collections were highly correlated to the venous collection with r^2 ^values ranging from 0.88 to 0.96 for fingerstick collection 1 and 0.93 to 0.96 for fingerstick collection 2.

**Table 3:** r^2 ^correlation values for fingerstick and venipuncture samples

	Fingerstick 1 vs. Fingerstick 2	Venipuncture vs. Fingerstick 1	Venipuncture vs. Fingerstick 2
**Donor 1**	0.94	0.88	0.94

**Donor 2**	0.97	0.95	0.94

**Donor 3**	0.97	0.96	0.96

**Donor 4**	0.97	0.96	0.95

**Donor 5**	0.97	0.95	0.93

### GeneChip Present/Absent Call Analysis

**Figure 3:**
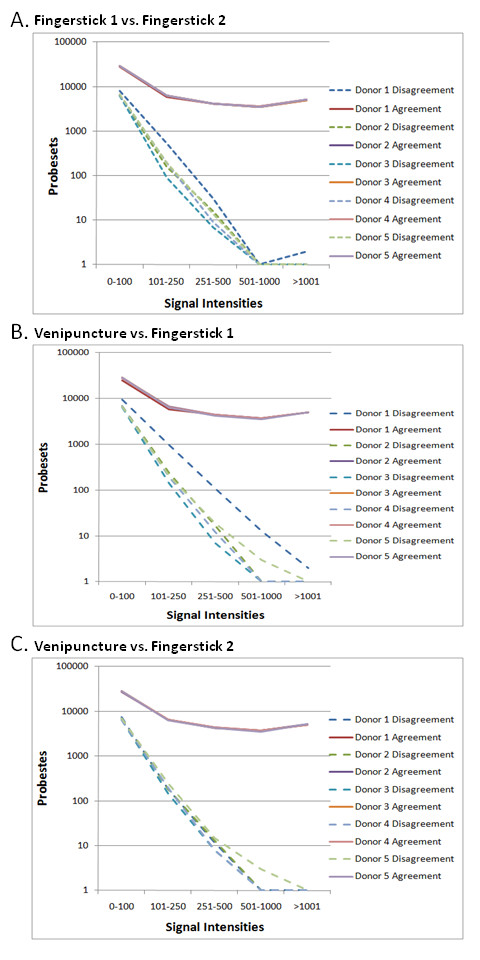
**Inverse correlations between signal intensities and number of disagreement calls**. The figures **A, B, and C **illustrate the inverse correlation between signal intensities and the number of disagreement calls; as the signal intensity increases, the disagreement calls between fingerstick samples and venipuncture samples drastically decreases.

We analyzed the degree of disagreement in Affymetrix present/absent calls between the fingerstick collections and also between the fingerstick and venous collections for each donor. Disagreement is described as a change in the present, marginal or absent calls between any two comparisons. Within each comparison we binned the average signal intensities of each probe set in the ranges between 0-100, 101-250, 251-500, 501-1000 and >1001. We got similar results for all three comparisons that we performed. There was an inverse correlation between the signal intensities and the disagreement calls for the probesets in each bin (Figure 3a, b &3c). In contrast, the number of probesets in agreement was not affected by the signal intensities irrespective of their bins, except at the lowest signal intensities (0-100).

We further analyzed the disagreement as the number of calls that changed from present to absent and vice versa to test the hypothesis that a change from absent to present would indicate higher sensitivity for the method used. On average, there was a higher number of disagreement calls, namely, change in call of a probe set from present in the venipuncture method to absent in the fingerstick method. However, there were also a number of calls that changed from present in the fingerstick collection to absent in the venipuncture collection (Table 4). For example, Donor 2 changed calls from present, in the venipuncture method, to absent, in the fingerstick method, at an average rate of 6.7%. On the other hand, Donor 2 changed calls from absent, in the venipuncture method, to present, in the fingerstick method, at an average rate of 4%.

**Table 4:** An analysis of the absolute change and percent change in calls between the fingerstick and venipuncture collections.

Venipuncture vs. Fingerstick 1	Donor 1	Donor 2	Donor 3	Donor 4	Donor 5
**P to A**	7704 (14.1%)	3826 (7.0%)	3581 (6.5%)	3725 (6.8%)	3030 (5.5%)

**A to P**	1359 (2.5%)	1988 (3.6%)	1786 (3.3%)	1894 (3.5%)	2553 (4.7%)

**Venipuncture vs. Fingerstick 2**	**Donor 1**	**Donor 2**	**Donor 3**	**Donor 4**	**Donor 5**

**P to A**	4248 (7.8%)	3486 (6.4%)	3139 (5.7%)	3395 (6.2%)	3054 (5.6%)

**A to P**	1921 (3.5%)	2360 (4.3%)	2167 (4.0%)	1960 (3.6%)	2716 (5.0%)

## Discussion

Despite the fact that technologies for RNA isolation have shown tremendous improvements over the past decade with RNA isolation kits for tissue, cells as well as whole blood, there is still no commercially available methodology for the isolation of good quality RNA from microliter volumes of human whole blood suitable for gene expression profiling on DNA microarrays. To test the hypothesis that we can isolate excellent quality and sufficient quantities of RNA from small volumes of whole blood (<100 μl) we investigated a modified protocol of whole blood RNA isolation in the present study.

Our results show that we were able to successfully isolate RNA that gave comparable results with the standard venous blood method when assayed on an Affymetrix GeneChip. The advantages of the fingerstick RNA isolation method were the ease of small volume blood collection, minimal modification of the standard PAXgene protocol, no requirement of a trained phlebotomist, and amenability to off-site studies as well as feasibility in studies involving serial blood collections on a large number of subjects. The fingerstick RNA was of very high quality, comparable to the venous blood RNA when assayed by the Agilent Bioanalyzer with no signs of degradation despite the technical differences between a venipuncture and a fingerstick collection. It is important to note that even though the volume of blood used in the fingerstick collection was small, the DNase treatment of the RNA was found to be crucial. This was especially necessary for the accurate quantitation of the RNA since the Nugen protocol calls for nanogram quantities of RNA as the starting material. The accurate quantitation of RNA could be biased due to the presence of contaminating DNA.

The yields of amplified cDNA were only slightly higher in the venipuncture method compared to both fingerstick collections. However, the Agilent Bioanalyzer traces for the amplified cDNA from the Nugen protocol showed no differences between the two collection methods. The quality control metrics for the GeneChip data showed that the venous collections had slightly higher average percent present calls (3.5% more) but this could reflect the inherent biological differences between venous and capillary blood. In a recent publication, Schalk et al., show that there are significantly higher WBC and RBC counts in capillary blood but lower numbers of platelets[22]. Such differences can contribute to altered gene expression in these two compartments of blood. In the design of any study this would not be a major influencing factor since cross-comparisons between two different methodologies and sources of samples is not very informative in gene expression studies.

On further analysis we showed that the differences are mainly due to the flux between calls at the lower signal intensities (<100), which accounts for almost all the variation between the methodologies. On average only about 12% of the total probesets on the GeneChip disagreed between any two comparisons that we made, which is well within the range of expected variation between samples in any DNA microarray study (data not shown). A study which looked at the individuality and variation in gene expression patterns in human blood found that there were several significant variations in gene expression among 77 samples of peripheral blood collected from normal healthy volunteers[23]. The variation was seen with respect to cellular and physiological themes, age, gender as well as temporal variations. However, the authors conclude that an analysis of multiple sequential samples from the same individuals allowed them to discern donor-specific patterns of gene expression. Two other similar studies investigating the variation in gene expression from the peripheral blood of healthy individuals also concluded that there are significant inter-individual differences in gene expression attributing them to genes that were involved in immunoglobulin class switching, interferon expression, X and Y-linked clusters, histone rich regions and killer cell function[24,25].

## Conclusion

In the present study we demonstrate a simple, modified RNA isolation protocol from small volumes of whole blood (70 μL by fingerstick) that is highly comparable to the standard method of RNA isolation (2.5 mL by venipuncture). The RNA from the fingersticks were further assayed on Affymetrix GeneChips and the results were very similar to the venipuncture collections. We effectively show that this fingerstick RNA isolation methodology can be used and should open up a broad range of applications for whole blood DNA microarray analysis ranging from pediatric studies to animal studies where there is access to only smaller volumes of blood. This methodology is especially suitable for serial monitoring, field testing, pharmacokinetic assays and rapid diagnosis of disease states.

## Methods

### Patient Recruitment

All samples were drawn from normal, healthy volunteers as part of The Scripps Research Institute's Normal Blood Drawing Service approved by the Office of Research Subjects Protection of the Scripps Health Human Subjects Committee. Both fingerstick and venous blood samples were collected by a trained phlebotomist.

### Sample collection

Sterile, DNase and RNase free, 1.5 mL Eppendorf tubes pre-filled with 200 μL PAXgene RNA stabilizing reagent (aliquoted from a PAXgene tube) were prepared and set aside. The donor finger was cleaned with an alcohol wipe and stuck with a single-use, spring loaded, retracting needle lancet (Unistick2 Super Lancet) according to the manufacturer's directions. Approximately 70 μl of blood was immediately collected into a capillary tube (Fisherbrand Heparinized Micro-Hematocrit). The sample was then transferred into the Eppendorf tube containing the PAXgene solution and the capillary tube was aspirated with a pipette to increase sample recovery. The sample was then mixed well with a pipette, given a quick spin, and left to incubate at room temperature; allowing complete lysis of blood cells in sample (PAXgene Blood RNA Kit handbook, http://www1.qiagen.com/Products/RnaStabilizationPurification/DSP/PaxGeneBloodRnaKitIVD.aspx#Tabs=t2[17].

### RNA Isolation and Purification

After incubation, we centrifuged the samples at 4500 g (Eppendorf Centrifuge Model 5804 R) at room temperature for 15 minutes. After carefully aspirating and discarding the supernatant, the pellet was washed with 1 mL RNase free water. From this point forward, we followed the processing guidelines given in the PAXgene Blood RNA kit (product# 762164) protocol. Note: As the pellet was often in the form of a streak on the back of the tube, we used the pipette tip to scrape it from the tube and fully re-suspend the pellet.

### RNA Yield, Purity and Integrity

Due to the small amount of total RNA in the eluate, the samples were dried in a SpeedVac (Thermo Scientific, Asheville, NC) to about 20 μL (from the standard 80 μL elution) before any quality assessment was done. The samples were then measured using the NanoDrop 1000 (NanoDrop, Wilmington, DE) to determine the concentration and purity. The Agilent 2100 BioAnalyzer and accompanying software was used to determine the integrity and quality of the total RNA.

### RNA Amplification, Labeling, and Fragmentation

50 ng aliquots were amplified using the Nugen Ovation^® ^RNA Amplification System V2 (Cat.# 3100) in conjunction with the Ovation^® ^WB Reagent (Cat.# 1300). They were subsequently purified using the Qiagen QIAquick^® ^PCR Purification Kit (Qiagen, Cat. #28104). Other purification methods can be used, according to the Nugen user guide, but we only tested the kit listed above. Again, the NanoDrop 1000 and Agilent 2100 BioAnalyzer were used to assess sample quantity and quality. The samples were then labeled and fragmented using the Nugen FL-Ovation™ cDNA Biotin Module V2 (Cat.# 4200).

### cDNA Hybridization and GeneChip Processing

All sample hybridization and GeneChip processing were done according to the Nugen standard array protocol for cDNA hybridization (Nugen user guide) and the standard Affymetrix protocols http://www.affymetrix.com/support/downloads/manuals/expression_analysis_technical_manual.pdf[21].

## Authors' contributions

Conceived and designed: DS, SK, AW, TM, ER, and SH. Performed experiments: SK, AW, ER, and TM. Analyzed data: SK, ER, TM, and AW. Wrote paper: SK, TM, and ER. Read and approved paper: DS, SK, AW, TM, ER, and SH.
